# A New Genus and Species of Syspastoxyelidae (Hymenoptera) from Mid-Cretaceous Burmese Amber †

**DOI:** 10.3390/insects17030289

**Published:** 2026-03-06

**Authors:** Xiao Li, Gengyun Niu, Meicai Wei

**Affiliations:** Laboratory of Insect Systematics and Evolutionary Biology, College of Life Sciences, Jiangxi Normal University, Nanchang 330022, Chinaniug@jxnu.edu.cn (G.N.)

**Keywords:** *Cilioxyela*, Symphyta, Mesozoic, Myanmar, Hukawng Valley, marginal setae

## Abstract

Syspastoxyelidae is an extinct basal hymenopteran lineage currently known only from mid-Cretaceous Burmese amber. Here, we describe a new genus and species, *Cilioxyela setosa* gen. et sp. nov., based on a well-preserved female specimen from the Hukawng Valley, northern Myanmar. The new taxon is assigned to Syspastoxyelidae based on diagnostic characters, including strongly proximally condensed forewing venation, a composite first flagellomere formed by fused ancestral segments, tibiae bearing dense robust spines, and segmented cerci. *Cilioxyela* gen. nov. differs from all previously described genera by a unique character combination, most notably, a distal forewing veinless membrane lacking longitudinal corrugation and conspicuously elongated marginal setae, together with a narrowed forewing, elongate pterostigma and anal cell, and distinctive antennal segmentation. These features support the establishment of a new genus. Comparative analysis indicates that distal forewing morphology in Syspastoxyelidae is more variable than previously recognized. The presence or absence of longitudinal corrugation in the distal forewing membrane likely reflects genus-level differentiation rather than a stable family-level synapomorphy. The new genus also supports a tentative division of Syspastoxyelidae into at least two morphologically cohesive groups, pending testing through additional fossil discoveries and quantitative phylogenetic analyses. The discovery of *Cilioxyela setosa* expands the known morphological disparity of Syspastoxyelidae and highlights evolutionary plasticity in distal forewing architecture among early Hymenoptera, contributing to a better understanding of morphological diversification in mid-Cretaceous forest ecosystems.

## Simple Summary

Syspastoxyelidae is an extinct group of early hymenopterans known only from mid-Cretaceous Burmese amber. Although rare, these fossils provide important information on the early morphological diversification of basal Hymenoptera. In this study, we describe a new genus and species, †*Cilioxyela setosa*, based on a well-preserved amber inclusion from northern Myanmar. The new taxon can be clearly assigned to Syspastoxyelidae based on key family characters but differs from all previously known genera by the combination of a smooth distal forewing membrane and the presence of conspicuously elongated marginal setae. Comparison with other syspastoxyelid genera suggests that distal forewing structures were more variable than previously assumed. This discovery expands the known morphological diversity of the family and provides new insights into the early evolutionary experimentation in wing structure among basal hymenopterans during the mid-Cretaceous.

## 1. Introduction

Syspastoxyelidae represents an extinct basal lineage of Hymenoptera currently known exclusively from mid-Cretaceous Burmese amber of the Hukawng Valley, northern Myanmar. The family was established relatively recently and presently comprises six genera and seven described species based on amber inclusions [1,2,3,4,5]. Syspastoxyelidae is characterized by a combination of morphological features typical of early diverging hymenopteran lineages, including a composite first flagellomere formed by fusion of ancestral flagellar segments, forewing venation strongly condensed toward the wing base, tibiae bearing dense robust spines, and segmented cerci [1,4]. This character complex distinguishes syspastoxyelids from other basal hymenopteran groups while simultaneously indicating their relatively early position within hymenopteran morphological diversification.

Since the establishment of the family based on *Syspastoxyela rhaphidia* by Engel et al. (2016), the discovery of additional Burmese amber inclusions has gradually expanded the morphological range recognized within the family [1]. Newly described taxa demonstrate appreciable variation in body size, forewing venational pattern, and leg morphology, while retaining the principal diagnostic character complex [2,3,4,5]. This combination of morphological stability at the family level and variability at the generic level is consistent with an early stage of lineage diversification and suggests that the evolutionary history of Syspastoxyelidae was more complex than initially assumed.

Among known morphological features, forewing structure is particularly characteristic of Syspastoxyelidae. The forewing typically shows a pronounced proximal concentration of venation combined with a distally veinless membrane. In most described taxa, the distal membrane bears longitudinal corrugations [1,2,3,4,5], which have been interpreted as a structural reinforcement mechanism [3,4]. Comparable reinforcement structures occur in several larger hymenopteran groups, including Siricidae [6]. However, in view of the small body size of syspastoxyelids (2.69–4.93 mm), these structures may reflect different functional or ecological constraints. Whether distal forewing membrane structure follows a single evolutionary trajectory within the family, and whether longitudinal corrugation represents a stable higher-level character or lineage-specific modification, remains insufficiently explored.

During examination of Burmese amber inclusions from the Hukawng Valley, we identified a well-preserved female specimen attributable to Syspastoxyelidae. The specimen exhibits the principal diagnostic characters of the family, including a composite first flagellomere, strongly proximally condensed forewing venation, tibiae bearing dense spines, and segmented cerci. At the same time, it differs from all previously described genera in possessing a distal forewing veinless membrane lacking longitudinal corrugation and bearing elongated marginal setae. This character combination has not been documented previously within the family.

Here, we provide a detailed morphological description and comparative analysis of this specimen and establish a new genus and species. The distinctive distal forewing structure of the new taxon provides additional data for evaluating morphological evolution and structural diversification within Syspastoxyelidae and contributes to the understanding of early hymenopteran diversification during the mid-Cretaceous.

## 2. Materials and Methods

Specimens were examined under a Motic SMZ-171 stereomicroscope (Motic, Xiamen, China). Specimen images were captured using a Leica Z16 APO microscope (Leica Microsystems, Wetzlar, Germany), focus-stacked with Helicon Focus (HeliconSoft 8.0, Kharkiv, Ukraine), and further processed in Adobe Photoshop 2021. The holotype (accession number: BMM-W1222) is permanently deposited in the Blue Miracle Museum Science Research Studio, Guangzhou, China. The amber originates from the Hukawng Valley, Tanai Township, Kachin State, northern Myanmar. U-Pb dating of zircons from the amber-bearing strata indicates an age of 98.79 ± 0.62 Ma [7,8]. The specimen was acquired in 2016 from a licensed amber dealer, prior to the escalation of armed conflict in the region in 2017, and its acquisition conforms to the recommendations of the International Palaeoentomological Society regarding the ethical study of Myanmar amber [9].

In this study, we followed the modified wing venation nomenclature proposed by Goulet and Huber [10]. The vein symbols used in our analysis include major longitudinal veins (e.g., Sc, R, Rs, Rs + M, M, Cu, and M+Cu). Sections of these veins are labeled as 1-Rs, 1-M, etc., while crossveins are designated as 1r-rs, 2r-rs, 2r-m, and related crossveins. We also used standardized terms for wing cells (e.g., 1R, 2Rs, 1M) to ensure consistency and clarity in interpretation.

## 3. Results

**Systematic palaeontology**

**Order Hymenoptera Linnaeus, 1758**

**Family** †**Syspastoxyelidae Engel and Huang, 2016**

**Genus** †***Cilioxyela* Li and Wei, gen. nov.**

**LSID:** urn:lsid:zoobank.org:act:D4AB82CD-20B5-49AF-AFCA-8B5C06DD666F

**Type species.** †*Cilioxyela setosa* gen. et sp. nov.

Etymology. The generic name *Cilioxyela* is formed from the Latin *cilium* (“fringe”) and *Xyela*, in reference to the presence of a conspicuous apical wing fringe. Gender: feminine.

**Diagnosis.** Frons broad and flattened; compound eyes large; gena narrow. Antennal thread composed of eight flagellomeres and distinctly shorter than the first flagellomere; second segment of antennal thread longer than remaining antennal thread segments. Pterostigma elongate and weakly sclerotised. Sc+R, from origin of Rs to proximal margin of pterostigma, slightly arched and positioned close to vein C. Cell 1M about 1.8× as long as high; Rs+M distinctly arched, forming a distinct angle at junction with 2-M. Rs distal to 2r-rs gently curved, terminating distinctly proximal to pterostigmal apex. Crossvein cu-a intersecting distal one-third of cell 1M and distal one-third of anal cell, cu-a inclined distally. Anal cell elongate, length exceeding twice width, and longer than cell 1M. Forewing distinctly narrowed; venation strongly shifted basally and largely confined to proximal half of wing; distal veinless membrane lacking longitudinal corrugation; wing margin fringed with setae, apical fringe longest, exceeding half of wing width, and gradually shortening laterally toward both anterior and posterior margins.

†***Cilioxyela setosa* gen. et sp. nov.**

**LSID:** urn:lsid:zoobank.org:act:DB1D5316-7FD0-4085-B596-D820061DD05A

**Etymology.** The specific epithet *setosa* is derived from the Latin adjective *setosus*, meaning “setose”, and refers to the conspicuously elongated apical fringe of setae along the wing margin.

**Locality and horizon.** The amber specimen was collected from Kachin (Hukawng Valley) in northern Myanmar, mid-Cretaceous.

**Diagnosis.** As for the genus (vide supra).

**Figure 1:**
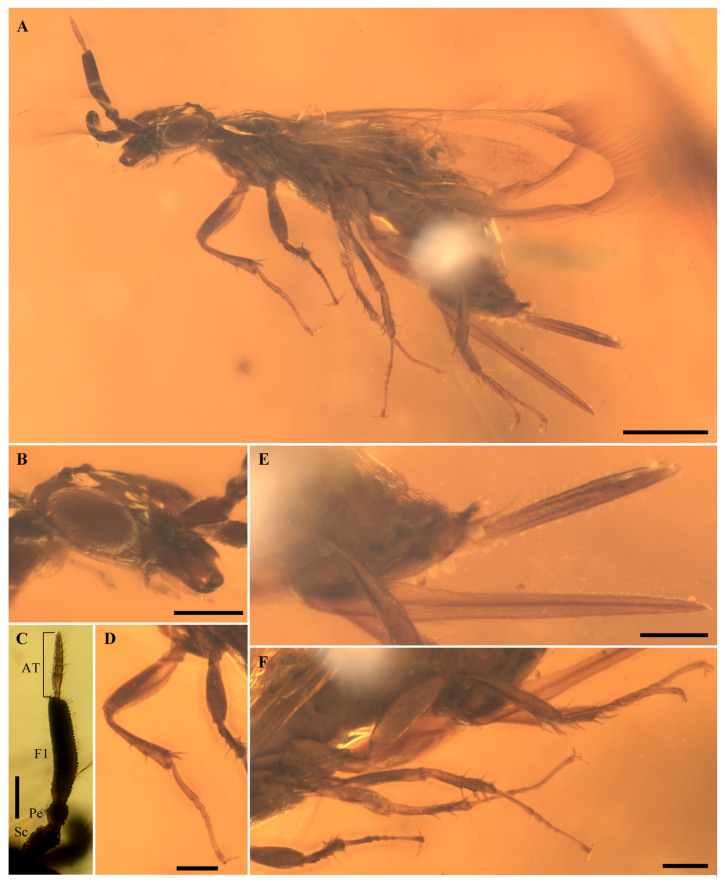
Photographs of *Cilioxyela setosa* gen. et sp. nov., holotype. (**A**) Lateral view. (**B**) Head. (**C**) Antenna. (**D**) Foreleg. (**E**) Ovipositor. (**F**) Midleg and hindleg. Abbreviations in (**C**): Sc, scape; Pe, pedicel; F1, first flagellomere; AT, antennal thread. Scale bars: 0.5 mm in (**A**); 0.2 mm in (**B**–**F**).

**Figure 2:**
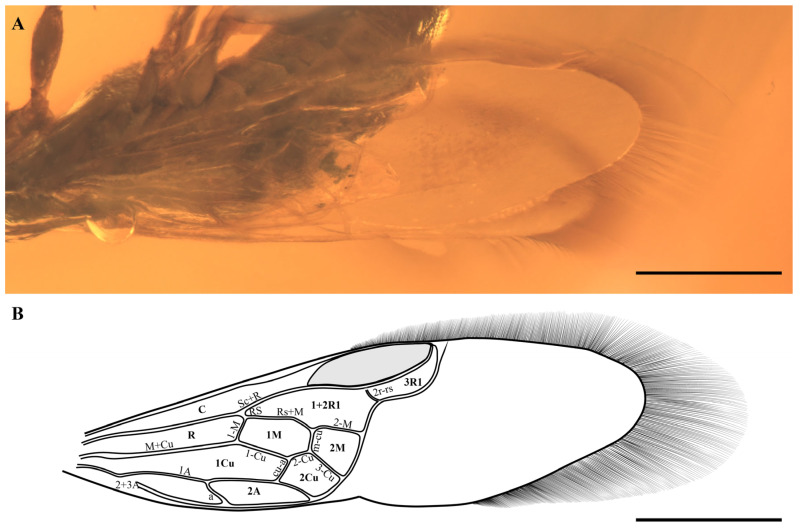
*Cilioxyela setosa* gen. et sp. nov., holotype. (**A**) Forewing. (**B**) Interpretative line drawing of the forewing’s venation, Scale bar: 0.5 mm.

**Holotype.** Female. BMM-W1222, deposited in the Blue Miracle Museum Science Research Studio, Guangzhou, 510000, China (Figure 1 and Figure 2).

**Measurements.** Total body length (as preserved, excluding ovipositor and antennae) 3 mm; antenna length 0.8 mm; forewing length (excluding marginal setae) ca. 2 mm, maximum width 0.6 mm; forewing marginal setae length approximately 0.42 mm; ovipositor length 1.2 mm.

**Head.** Head large and broad; compound eyes strongly elongate and moderately convex; gena narrow; ocelli slightly elevated, situated close to the occipital margin and widely separated from the antennal socket; frons elongate, flattened and broad, with a distinct longitudinal carina along its anterior portion; clypeus short and straight; mandible elongate, with a single large apical tooth observable; maxillary palp distinctly longer than labial palp (Figure 1B). The antenna is 11 segmented in total, consisting of scape, pedicel, a composite first flagellomere formed by fusion of multiple ancestral flagellomeres, and an antennal thread composed of eight distal flagellomeres; scape elongate, about 2× as long as wide, base narrower than apex; pedicel short, as long as wide; first flagellomere greatly elongate and thickened, about 5.0× as long as wide; antennal thread distinctly shorter than the first flagellomere, slender at the base and apex; second flagellomere short, approximately as long as wide; third flagellomere the longest of the flagellomeres except for the first; subsequent flagellomeres progressively shorter and narrower apically, with the terminal flagellomere very small, slightly shorter than the second flagellomere (Figure 1A,C).

**Thorax.** Thorax moderately robust, appearing slightly arched in lateral view. Pronotum short and transverse, with an inclined anterior slope; mesoscutum with both prescutum and scutum distinctly convex; notauli present and well defined; mesepisternum bearing a transverse sulcus; mesoscutum and mesoscutellum largely obscured by overlapping wings and legs, thus not clearly discernible (Figure 1A).

**Legs.** Legs slender and elongate (Figure 1D,F). Fore coxae moderately developed, whereas hind coxae relatively enlarged; trochanters of the mid and hind legs relatively elongate; femora robust, slightly clavate; all tibiae armed with strong spines dorsally; fore, mid, and hind tibiae each with two apical spurs; tarsi slender and elongate, tarsus longer than femur and tibia, tarsomeres II–IV short and subequal in length, their combined length distinctly shorter than that of the basitarsus; fifth tarsomere elongate but slightly shorter than the basitarsus, pretarsal claws long, with curved apices and minute subapical tooth, individual claw more than one-half length of fifth tarsomere.

**Abdomen.** Abdomen elongate and slender; abdominal segmentation and surface sculpture indistinct due to preservation. Ovipositor long, exceeding half the length of the abdomen; ovipositor sheath separated from lancet and lance, flattened; apical sheath slightly longer than basal sheath; lancet and lance sword-shaped; cercus with two discernible segments; basal cercal segment short and broad, apical segment slightly narrower than the basal one and about 1.5× as long, bearing slender apical setae (Figure 1E).

**Wings.** Forewing (Figure 2A,B) distinctly narrowed; venation strongly shifted basally and largely confined to the proximal half of the wing, wing distinctly constricted at approximately the distal one-fifth of the wing length, the distal veinless membrane smooth; wing margin fringed with setae, the apical fringe being the longest, exceeding half of the wing width, and gradually shortening laterally toward both the anterior and posterior margins. All veins tubular and lightly pigmented; all cells closed. Costal cell wide; Sc completely fused with R, forming Sc+R; Sc+R straight except for a slight angulation at the origin of Rs and weakly arched toward the distal margin of the pterostigma; pterostigma elongate and weakly sclerotised; 1-Rs much shorter than 1-M; Rs+M junction slightly angled; cell 1M about 1.8× as long as high; Rs+M distinctly arched distally, forming a conspicuous angle at its junction with 2-M; 2r–rs present, positioned anterior to the middle of the pterostigma; cell 3R1 long, with Rs closely following the pterostigmal margin to form a narrow, arched cell; Rs not bifurcate apically, terminating distinctly basal to the pterostigmal apex; crossvein cu-a intersecting the distal one-third of cell 1M and also intersecting the distal one-third of the anal cell, cu-a inclined distally. Anal cell elongate, with a length-to-width ratio exceeding 2, and longer than cell 1M. Hind wing not observable due to preservation.

**A key to known genera and species of** the family †**Syspastoxyelidae.**

1. Antennal thread fewer than 12 segmented, distinctly shorter than the first flagellomere, flagellar segments non-uniform; Rs reaching wing margin distinctly distant from the pterostigmal apex; the distal veinless membrane without longitudinal corrugation...................................................................................................................................**2**

– Antennal thread 12 segmented, not shorter than the first flagellomere, flagellar segments uniform; Rs reaching wing margin at or near the pterostigmal apex; the distal veinless membrane with longitudinal corrugation ...........................................................**3**

2. Forewing broad; pterostigma short and wide; cu-a inclined anteriorly; anal cell shorter than cell 1M; forewing margin without distinct long marginal setae ........................................................***Syspastoxyela rhaphidia* Engel and Huang, 2016**

– Forewing narrowed; pterostigma elongate and relatively narrow; cu-a inclined distally; anal cell longer than cell 1M; forewing margin with distinct long marginal setae ...........................................***Cilioxyela setosa* gen. et sp. nov.**

3. Pterostigma weakly sclerotised; costal area narrow; 2r–rs positioned near proximal one-third of pterostigma..........***Pinguixyela pinguis* (Wang, Shih, Ren and Gao, 2019)**

– Pterostigma sclerotised; costal area wide; 2r-rs near midlength of pterostigma or more distal ..............................................................................................................................**4**

4. Vein 1-Rs extremely short, dot-like; 4-Rs fully developed and strongly curved; cell 3R1 wide; cell 1M with length-to-width ratio exceeding 1.5; mid femur almost disc-like .................................................................***Burmoxyela lii* Zheng and Rasnitsyn, 2021**

– Vein 1-Rs short but petiolate; 4-Rs slightly curved; cell 3R1 narrow; cell 1M with length-to-width ratio distinctly less than 1.5; mid femur elongate .................................**5**

5. ***Pronotum*** strongly elongate, triangular, with angular apex; r–m distinctly shorter than 2-M ............................................***Deltoxyela engeli* Wang, Shih, Ren and Gao, 2019**

– Pronotum less elongate; r–m as long as or longer than 2-M ...........................................**6**

6. ***Pterostigma*** narrow; Rs reaching wing margin only slightly beyond pterostigmal apex ............................................................................***Grandixyela rasnitsyni* Zheng, 2021**

– Pterostigma wide; Rs reaching wing margin before pterostigmal apex................................***Striaexyela* Zheng, 2019** .............................................................. **7**

7. Vein 2r–rs about half length of 4-Rs; r–m distinctly longer than 2-M; 2m–cu convex, more than 2× length of 2-M ....................................***Striaexyela longicornis* Zheng, 2019**

– Vein 2r–rs not exceeding 0.3× length of 4-Rs; r–m subequal to 2-M; 2m–cu straight, about 1.5× length of 2-M ..............***Striaexyela simpla* (Wang, Shih, Ren and Gao, 2019)**

## 4. Discussion

### 4.1. Systematic Position of the New Genus Cilioxyela Within Syspastoxyelidae

The placement of *Cilioxyela* gen. nov. within Syspastoxyelidae is supported by a suite of morphological characters. These include strongly proximally condensed forewing venation, a composite first flagellomere formed by the fusion of multiple ancestral flagellomeres, tibiae densely armed with robust spines, and segmented cerci—features that are diagnostic of the family Syspastoxyelidae and consistent with the placement of *Cilioxyela* within this family [1,2,3,4,5].

*Cilioxyela* gen. nov. is distinguished from other syspastoxyelids by the following combination of characters, including a broad and flat frons, relatively large compound eyes, an 11 segmented antenna, a distinctly narrowed forewing, an elongate and relatively narrow pterostigma, vein cu-a inclined distally, an anal cell longer than cell 1M, the distal veinless membrane smooth; wing margin fringed with setae, and wing distinctly constricted at approximately the distal one-fifth of the wing length. This character combination is not observed in any previously described genus of Syspastoxyelidae and is clearly expressed in the well-preserved holotype specimen, providing robust justification for the recognition of *Cilioxyela* as a distinct genus.

The antennal morphology of *Cilioxyela* gen. nov. is overall most similar to that of *Syspastoxyela* and clearly differs from that observed in the remaining known genera of Syspastoxyelidae. In both taxa, the antennal thread is distinctly shorter than the composite first flagellomere and shows a relatively uniform diameter from base to apex, with only slight narrowing distally. In *Cilioxyela*, the first segment of the antennal thread is extremely short and is followed by a distinctly elongate second segment. Based on this configuration, a comparable pattern of flagellar segmentation may be inferred for *Syspastoxyela*.

In *Syspastoxyela*, the junction between the composite first flagellomere and the antennal thread is markedly curved. In available specimens and published illustrations, the first clearly visible segment of the antennal thread appears elongate. This condition may plausibly reflect the presence of an additional, extremely short basal flagellar segment positioned between the composite first flagellomere and the elongate segment. However, given the limitations imposed by preservation and viewing angle in available specimens, this interpretation should be regarded as tentative. Furthermore, careful examination of the apical portion of the antennal thread in *Syspastoxyela* suggests that it is likely composed of two segments, with the terminal segment being minute and therefore easily overlooked. Taken together, the inferred presence of a concealed basal segment and a minute apical segment raises the possibility that the antennal thread may comprise more segments than previously reported.

With the familial placement of *Cilioxyela* firmly established, comparative evaluation of its morphology further provides insights into higher-level systematic differentiation within Syspastoxyelidae. Based on currently available material, *Cilioxyela* and *Syspastoxyela* share a set of morphological features, including an antennal thread composed of less than 12 segments and shorter than the composite first flagellomere, with non-uniform flagellar segments; Rs terminating at the wing margin distinctly basal to the pterostigmal apex, a gently arched outermost forewing venation, and the distal veinless membrane without longitudinal corrugation [1].

In contrast, *Burmoxyela*, *Deltoxyela*, *Grandixyela*, *Pinguixyela*, and *Striaexyela* display a different, internally consistent character suite, including a longer antennal thread (typically with 12 segments) exceeding the length of the first flagellomere, relatively uniform flagellar segments, Rs terminating near or at the pterostigmal apex, a sharply angled and shortened outermost forewing venation, and the distal veinless membrane usually bearing distinct longitudinal corrugation [2,3,4,5].

Based on these differences in character combinations, it is tentatively suggested that Syspastoxyelidae may comprise at least two morphologically cohesive groups, with *Cilioxyela* and *Syspastoxyela* forming one group and the remaining genera constituting another. Given the limited number of available specimens, this proposal should be regarded as preliminary and subject to testing through the discovery of additional well-preserved material and the application of formal phylogenetic analyses based on quantitative morphological character matrices. Nevertheless, this phenetic framework offers a useful working hypothesis for future discussions of tribal-level morphological differentiation within Syspastoxyelidae.

### 4.2. Evolutionary and Ecological Implications of Distal Forewing Morphology

The taxonomic significance of longitudinal corrugations in the veinless membrane of the forewing has gradually gained taxonomic attention within Syspastoxyelidae. In the original description of *Syspastoxyela rhaphidia*, Engel et al. (2016) did not mention such structures, and the published images do not clearly show longitudinal corrugation in the distal forewing [1]. Subsequently, Zheng et al. (2019), in their description of *Striaexyela longicornis*, explicitly documented pronounced longitudinal corrugations in the veinless membrane of the forewing and suggested that similar structures might also occur in *Syspastoxyela* but may have been overlooked due to observational limitations [3]. Thereafter, several genera and species, including *Deltoxyela engeli*, *Pinguixyela pinguis*, and *Grandixyela rasnitsyni*, were reported to possess such corrugations; in *Striaexyela simpla*, these structures are at least clearly observable in the hind wing [2,4,5].

In the newly described *Cilioxyela setosa*, the distal veinless membrane of the forewing is smooth, without longitudinal corrugations. When considered together with the original description and published imagery of *Syspastoxyela rhaphidia*, which likewise do not show clear evidence of longitudinal corrugation [1], and given the overall similarity between these two genera in wing venation and antennal morphology, current evidence is most consistent with the interpretation that variation in the distal veinless membrane, whether smooth or bearing longitudinal corrugation, is more likely to reflect genus-level differentiation rather than a stable, family-level synapomorphy of Syspastoxyelidae. The diagnostic stability of this character at the family level, therefore, remains to be evaluated based on additional well-preserved material.

Based on currently available fossil evidence, variation in the structure of the veinless membrane in Syspastoxyelidae can be provisionally categorized into at least three morphological types. The first type, observed in *Syspastoxyela rhaphidia*, is without longitudinal corrugation [1], possibly representing a relatively simplified condition. The second type, present in most known genera of the family, is characterized by the distal veinless membrane with longitudinal corrugation. Comparable corrugations are also known in several relatively large hymenopteran groups, such as Siricidae, Psedosiricidae, and Scoliidae [3,6,11], where they have been generally interpreted as contributing to increased flexural stiffness and structural stability of the wing and potentially influencing local airflow during flapping flight [12,13,14]. The third type, represented by *Cilioxyela setosa*, is without longitudinal corrugation but exhibits conspicuously elongated marginal setae along the wing margin, forming a distal wing membrane configuration distinct from corrugation-based reinforcement. Within Hymenoptera, well-developed marginal setae are most characteristically found in extremely small-bodied groups such as Chalcidoidea (e.g., Mymaridae; Jin 2016) [15], where they are typically associated with low-Reynolds-number flight conditions and may influence aerodynamic properties of the wing by modifying boundary-layer airflow [16,17]. Given the substantially larger body size of *Cilioxyela* compared with typical setose-winged microhymenopterans, this functional interpretation should be regarded as a tentative morphology–function hypothesis rather than a definitive conclusion.

From a phylogenetic perspective, the distribution of distal forewing morphologies within Syspastoxyelidae does not unambiguously indicate a single evolutionary pathway. Under the principle of parsimony, one conservative interpretative scenario is that the absence of longitudinal corrugation—observed in *Cilioxyela setosa* and apparently also present in *Syspastoxyela rhaphidia*—represents the plesiomorphic condition within Syspastoxyelidae, whereas longitudinal corrugation evolved secondarily as a derived form of structural reinforcement in certain lineages. Alternatively, another possible evolutionary pathway involves the retention of a non-corrugated distal veinless membrane combined with the development of conspicuously elongated marginal setae along the wing margin, resulting in a distal forewing configuration distinct from corrugation-based reinforcement, as exemplified by *Cilioxyela setosa*. This suggests that, against a background of strongly simplified wing venation, different genera of Syspastoxyelidae may have adopted multiple morphological strategies to meet the functional demands imposed on the distal wing membrane.

The diversity observed in distal forewing structures further indicates that the veinless membrane of the forewing in Syspastoxyelidae exhibits a certain degree of evolutionary plasticity. Such plasticity was likely shaped by the combined influence of body size, flight behaviour, and microhabitat use, and may have had direct consequences for flight performance and activity patterns under specific environmental conditions [18,19]. Consequently, variation in the structure of the distal veinless forewing carries not only taxonomic significance but also provides valuable insight into potential ecological differentiation among genera within the family.

In addition to the prominent setae of the forewing margin, *Cilioxyela setosa* exhibits several other morphological features potentially related to ecological adaptation. Its compound eyes are relatively well developed, occupying a larger proportion of the head capsule than in some other syspastoxyelid genera. Studies of extant insects suggest that such traits are often associated with activity under low-light conditions or adaptation to visually complex environments [20,21,22]. Moreover, as in other members of Syspastoxyelidae, the tibiae of *Cilioxyela setosa* bear multiple spurs accompanied by dense and robust spines, a configuration generally interpreted as facilitating locomotion on structurally complex substrates such as tree trunks, branches, or bark crevices [2,23,24]. Combined with pollen grains preserved in the abdominal region of *Syspastoxyela rhaphidia* amber specimens, which indicate ecological associations with coniferous plants [1], these observations suggest that *Cilioxyela setosa* may have preferentially occupied the surfaces of gymnosperms (e.g., conifers) or inhabited relatively closed forest microhabitats. Its ecological niche may therefore have differed to some extent from that of other syspastoxyelid genera.

In summary, Syspastoxyelidae exhibits notable genus-level morphological diversity in Burmese amber, with particularly pronounced variation in distal forewing structures. Comparable patterns of diversification are also observed in other basal hymenopteran groups from the same deposit, such as Anaxyelidae, suggesting that this phenomenon may reflect broader regional evolutionary trends. The West Burma Terrane, which is inferred to have experienced prolonged island isolation during the mid-Cretaceous [25,26,27], may have provided relatively independent evolutionary settings conducive to morphological differentiation and specialization in these basal lineages.

## 5. Conclusions

Based on a well-preserved hymenopteran fossil from mid-Cretaceous Kachin amber of northern Myanmar, this study establishes a new genus and species, *Cilioxyela setosa* gen. et sp. nov., within the extinct family Syspastoxyelidae. The new taxon conforms closely to the family-level diagnostic features of Syspastoxyelidae, including markedly basally condensed forewing venation, a strongly fused first flagellomere, and tibiae bearing dense, robust spines, segmented cerci, supporting its attribution to this family. At the same time, *Cilioxyela setosa* is distinguished from all previously known genera by a unique and consistent combination of characters, most notably the absence of longitudinal corrugation in the distal forewing membrane combined with the presence of well-developed marginal setae, as well as several consistent differences in wing and antennal morphology. These features together provide a reliable basis for generic distinction.

The discovery of *Cilioxyela setosa* adds to the known generic diversity of Syspastoxyelidae and suggests that the distal forewing architecture in this family may have exhibited a broader range of morphological variation than previously recognised. The presence or absence of longitudinal corrugation in the forewing membrane is more likely to reflect genus-level morphological differentiation within Syspastoxyelidae than a stable family-level synapomorphy, although this interpretation requires further evaluation based on additional well-preserved fossil material. The continued discovery of new amber inclusions and their incorporation into comparative and phylogenetic analyses will likely refine understanding of the systematic position of Syspastoxyelidae within early Hymenoptera, the evolutionary pathways of their morphological innovations, and their possible ecological associations with Mesozoic plant resources.

## Data Availability

The original contributions presented in this study are included in the article. The supplementary photographs and original Z-stacks of the holotype specimen *Cilioxyela setosa* gen. et sp. nov. (BMM-W1222) are available from Zenodo at https://doi.org/10.5281/zenodo.18872431. Further inquiries can be directed to the corresponding author.
